# Psychophysical changes after total sleep deprivation and experimental muscle pain

**DOI:** 10.1111/jsr.14329

**Published:** 2024-09-17

**Authors:** Emma Hertel, Elaxmi Sathiyalingam, Linea Pilgaard, Simone Juline Brommann, Rocco Giordano, Kristian Kjær‐Staal Petersen

**Affiliations:** ^1^ Faculty of Medicine Aalborg University Aalborg Denmark; ^2^ Mathemathical Modeling of Knee Osteoarthritis (MathKOA) Aalborg University Aalborg Denmark; ^3^ Center for Neuroplasticity and Pain (CNAP) Aalborg University Aalborg Denmark; ^4^ Department of Oral and Maxillofacial Surgery Aalborg University Hospital Aalborg Denmark

**Keywords:** acute pain, interleukin 6, muscle injury, musculoskeletal pain, psychology, sensitisation

## Abstract

Sleep disturbances exacerbate chronic pain, increase psychological load, and increase inflammation. Delayed onset muscle soreness (DOMS) mimics aspects of chronic pain, predominantly affecting peripheral pain mechanisms, while experimental sleep provocations have been shown to impact central pain mechanisms. This study aimed to combine a DOMS model with total sleep deprivation (TSD) to create a novel model affecting both peripheral and central pain mechanisms. A total of 30 healthy participants attended two sessions (baseline and follow‐up) separated by 24 h of TSD and a home rating after 48 h. Assessments of interleukin 6 (IL‐6) levels, sleep quality, pain catastrophising, affect, and symptoms of depression and anxiety were included in the baseline and follow‐up sessions. Additionally, pressure pain and tolerance thresholds, temporal summation, and conditioned pain modulation (CPM) were assessed using cuff‐pressure algometry in the baseline and follow‐up sessions. DOMS was induced with eccentric calf raises during the baseline session followed by 24 h of TSD. At follow‐up pain tolerance (*p* = 0.012) was significantly reduced, and CPM (*p* = 0.036) was significantly impaired compared to baseline. Psychological changes included decreases in pain catastrophising (*p* = 0.027), positive affect (*p* < 0.001), negative affect (*p* = 0.003), and anxiety (*p* = 0.012). Explorative regression models predicted 58% and 68% of DOMS pain intensity after 24 and 48 h, respectively, based on baseline body mass index, pain thresholds, psychological measures, and IL‐6 (*p* < 0.01). Combining DOMS with 1 night of TSD induced pain hypersensitivity, impaired CPM, and altered psychological states. A combination of baseline inflammation, psychological measures, and pain sensitivity significantly predicted DOMS pain intensity after 24 and 48 h.

## 1 INTRODUCTION

Musculoskeletal pain is estimated to affect a fifth of the world population (Cieza et al., 2020) and is associated with considerable impacts on quality of life (Reid et al., 2011). Pain and poor sleep are common co‐occurrences (Sun et al., 2021) and approximately three‐quarters of patients with chronic pain have self‐reported poor quality of sleep (Sun et al., 2021). Short‐term insufficient sleep has been associated with short‐term manifestations of pain, stress, depression, and anxiety (Medic et al., 2017), while long‐term sleep problems can result in inflammation, dyslipidaemia, and hypertension (Medic et al., 2017). In patients with chronic pain, poor quality of sleep is associated with increased pain intensity (Morin et al., 1998), spreading of pain (Wiklund et al., 2020), and pain‐related disability (Naughton et al., 2007). Sleep and pain share several biological pathways, through which disruption of one can affect the other (Finan et al., 2013), creating a vicious cycle of mutual exacerbation. Supporting this, sleep provocations have been shown to increase pain sensitivity (Simpson et al., 2018; Smith et al., 2007; Staffe et al., 2019), while sleep extension can increase pain tolerance (Roehrs et al., 2012). Furthermore, poor sleep has been found to be predictive of pain development during a 5‐year period in pain‐free individuals (Aili et al., 2015).

Quantitative sensory testing (QST) can assess pain mechanisms and has previously been demonstrated to explain pain intensity (Arendt‐Nielsen et al., 2015; Kristensen et al., 2021) and predict treatment effects (Hertel et al., 2024). Common changes in patients with chronic musculoskeletal pain are facilitated temporal summation of pain (TSP) and impaired conditioned pain modulation (CPM), both human proxy assessments of central pain processing (Nir & Yarnitsky, 2015). Furthermore, several inflammatory substances fluctuate with the circadian rhythm exerting somnogenic or sleep‐inhibitory effects (Zielinski & Krueger, 2011) and experimental sleep disturbances are associated with increased serum levels of inflammatory substances (Irwin et al., 2016). Several inflammatory substances are known to sensitise pain mechanisms, and, thus, deficient sleep might increase pain sensitivity (Kawasaki et al., 2008). Finally, several indicators of negative affect such as depression, anxiety, and fear are associated with an increased risk of chronic musculoskeletal pain (Martinez‐Calderon et al., 2020). Poor quality of sleep has previously been found to be associated with increased pain catastrophising in patients with knee osteoarthritis (Boye Larsen et al., 2021). Increased positive affect has been associated with reduced pain severity (Ong et al., 2020) and suggested to facilitate adaptive coping to chronic pain by buffering maladaptive pain‐related cognitions leading to increased resilience and self‐management (Finan & Garland, 2015). Positive affect has previously been demonstrated to be predictive of acute muscle pain intensity (Kristensen et al., 2021).

Experimental models of pain mimicking features of chronic pain are useful to investigate factors relevant to pain development (Petersen et al., 2019). They enable research on the complex relationship between changes in pain sensitivity, sleep, and psychological factors by experimentally provoking these changes in healthy individuals. Delayed onset muscle soreness (DOMS) mimics aspects of musculoskeletal pain with severity varying from stiffness and mild‐to‐severe pain and restricted movement (Cheung et al., 2003). Still, DOMS models do not mimic systematic inflammation and psychological changes, which are often seen in patients with chronic musculoskeletal pain. Therefore, DOMS models are not necessarily a good experimental model of musculoskeletal pain. On the other hand, sleep provocations have been consistently shown to cause changes in central pain mechanisms (Hertel et al., 2023; Smith et al., 2007; Staffe et al., 2019) along with psychological changes (Finan et al., 2017; Hertel et al., 2023; Stenson et al., 2021; Talbot et al., 2010). Furthermore, total sleep deprivation (TSD) has recently been indicated to decrease pressure pain detection thresholds (PPTs) after DOMS (Palsson et al., 2023). Combining the peripheral acting components of the DOMS model with the central and systemic components of TSD could therefore provide additive effects, as TSD is believed to attenuate central sensitivity to pain and alter psychological state. This approach might enable a new translational framework by introducing a multiple‐hit model, which is suggested to be more effective for mimicking complex disorders (Kim et al., 2020). Therefore, the present study aimed to investigate pain sensitivity, inflammation, and psychological state before and after a DOMS and TSD model. Additionally, the present study aimed to investigate if baseline parameters could predict pain following DOMS to investigate whether certain subjects are more vulnerable to an acute painful injury. The primary outcome studied was CPM, and the primary endpoint was a tentative change in CPM from baseline and after intervention. It was hypothesised that the intervention would impair CPM due to a potential increase in inflammation, increase in general pain sensitivity and worsening of psychological factors.

## 2 METHODS

**FIGURE 1:**
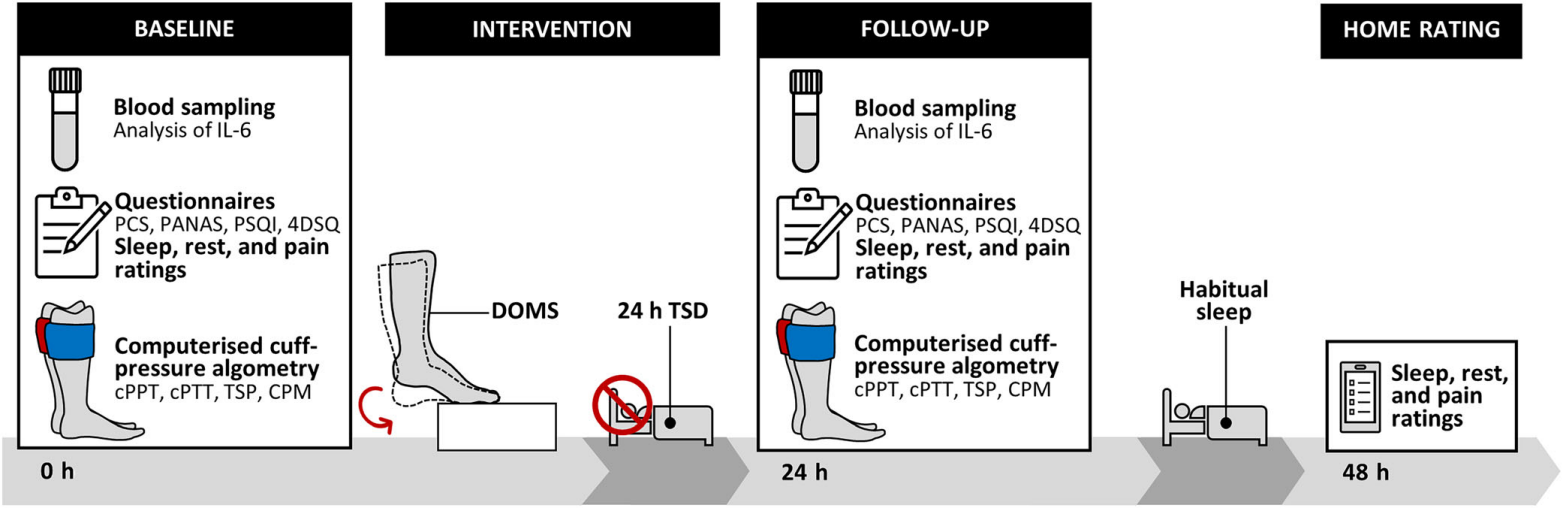
Overview of the experimental design. Both baseline (0 h) and follow‐up (24 h) include blood sampling, questionnaires, computerised cuff‐pressure algometry, and queries about sleep, rest, and DOMS pain. The intervention consists of DOMS induction in the index calf and 24 h of TSD. After the follow‐up (24 h) the participants will have 1 night of habitual sleep and then rate their sleep, rest and DOMS pain again. CPM, conditioned pain modulation; cPPT, cuff pressure pain detection threshold; cPTT, cuff pain tolerance threshold; DOMS, delayed onset muscle soreness; 4DSQ, Four‐Dimensional Symptom Questionnaire; IL‐6, interleukin 6; PANAS, Positive and Negative Affective Schedule; PCS, Pain Catastrophizing Scale; PSQI, Pittsburgh Sleep Quality Index; TSD, total sleep deprivation; TSP, temporal summation of pain.

Each participant attended two experimental sessions, separated by 24 h of TSD, and one home rating distributed 48 h after the baseline session. Both experimental sessions included blood testing, validated questionnaires, and cuff‐pressure algometry. In the baseline session (0 h) DOMS was induced. Participants then completed TSD at home whereafter they returned for the follow‐up session (24 h). Blood testing, cuff‐pressure algometry, and DOMS were administered by the study personnel while the questionnaires and sleep intervention were self‐administered. The home rating was distributed after 1 night of habitual sleep (48 h after the first session). An overview of the experimental protocol can be seen in Figure 1.

### 2.1 Participants

A total of 30 healthy participants aged 18–45 years were recruited from Aalborg University, Denmark, and wider community in April and May 2023 through notices on community boards and social media. Exclusion was warranted if they reported any of the following: drug addiction; previous or current neurological, musculoskeletal, pulmonary, cardiac, or chronic pain conditions as well as mental illnesses; current use of medications that might affect the study; recent history of acute pain; participation in other pain trials throughout the study period; or lack of cooperation.

### 2.2 Questionnaires

The participants answered four validated questionnaires at baseline (0 h) and follow‐up (24 h). The Pittsburgh Sleep Quality Index (PSQI) measures the subjective quality of sleep within the past month, summarised as a single score, with higher scores denoting worse quality of sleep (Buysse et al., 1989). The Pain Catastrophizing Scale (PCS) evaluates thoughts and feelings experienced during pain, with higher scores denoting an increased tendency to catastrophise (Sullivan et al., 1995). The Positive and Negative Affective Schedule (PANAS) measures positive and negative affective states during the past week, with high positive scores reflecting a high energy state with engagement, while high scores on the negative scale denote a state of lethargy (Watson et al., 1988). The Four‐Dimensional Symptom Questionnaire (4DSQ) measures symptoms of somatisation, anxiety, depression, and distress in the past week, with higher scores denoting increased severity (Terluin et al., 2014).

Finally, the participants rated their subjective quality of sleep during the previous night (0 ‘worst quality of sleep imaginable’ and 100 ‘best quality of sleep imaginable’), current level of rest (0 ‘not rested/refreshed at all’ and 100 ‘most rested/refreshed possible’), and current DOMS pain (0 ‘no pain’ and 100 ‘worst pain imaginable’) at baseline (0 h), follow‐up (24 h), and the home rating (48 h). All questionnaires were completed online with support from the study personnel available at baseline (0 h) and follow‐up (24 h), while the home rating was completed remotely.

### 2.3 Computerised cuff‐pressure algometry

A cuff‐pressure algometer (Cortex Technology, Aalborg University) with two inflatable air‐cuffs (VBM Medical) and an electronic visual analogue scale sampling at 10 Hz (eVAS; 0 ‘no pain’ and 10 ‘worst pain imaginable’) was used to investigate measures of pain sensitivity. The cuffs were positioned on the widest part of the calves and the dominant leg was chosen as the index leg.

The cuff PPT (cPPT) and cuff pain tolerance threshold (cPTT) were determined with a ramped inflation rate of 1 kPa/s performed on the index leg. The cPPT was defined as the kPa pressure when the eVAS reached 1 and the cPTT as the pressure when the ramp was stopped, either by the participant or reaching the safety cut‐off at 100 kPa. Temporal summation of pain (TSP) was assessed with 10 repeated pressure stimulations at 0.5 Hz intervals on the index leg at the cPTT level. Participants rated the initial pain and adjusted for the subsequent stimulations without returning to zero. TSP was calculated as the difference between the averaged first four inflations and the averaged final three, with a positive value denoting facilitation. CPM was determined by applying a conditioning stimulus at 70% cPTT on the contralateral leg, simultaneous with a 1 kPa/s ramp on the index leg. Participants rated only the ramped inflation on the eVAS. The CPM effect was determined as the difference in the cPPT with and without conditioning, with a positive value denoting functioning CPM.

### 2.4 Inflammatory analysis

At baseline (0 h) and follow‐up (24 h) whole blood samples (9 mL) were collected through venipuncture into EDTA tubes. Plasma isolation was conducted through centrifugation at 2000*g* for 15 min. The obtained plasma was stored in a −80°C freezer until analysis. Plasma was analysed for interleukin 6 (IL‐6) using the ProQuantum Immunoassay Human IL‐6 Kit (ThermoFisher Scientific) following the manufacturer's instructions. The test utilised proximity ligation assay technology, combining the analyte specificity of high‐affinity antibody–antigen binding with signal detection and amplification of real‐time polymerase chain reaction (QiAquant96; QIAGEN) for protein quantification allowing <0.05 pg/mL sensitivity. Each reaction was conducted in duplicates using 2‐μL plasma.

### 2.5 Intervention

The intervention consisted of a combination of DOMS of the index calf and a 24‐h TSD protocol.

The DOMS was induced on the index leg with four sets of 30 unilateral calf raises. Participants executed 1‐s raises and then lowered the heel over 2 s. Those unable to complete the 30 repetitions proceeded until failure. The pain was assessed during flexion immediately following the induction, at follow‐up (24 h), and the home rating (48 h). TSD was induced by revoking sleep for at least 24 h between baseline (0 h) and follow‐up (24 h). Adherence was controlled using wrist actigraphy (Fitbit Charge 4; Fitbit Inc.) and emails sent by the participant to the research team every 45 min throughout the night containing an image of the Fitbit watch face showing the corresponding time. In case any emails were missed compliance was checked using the actigraphy data.

### 2.6 Statistics

The primary outcome of the present study was a change in CPM from baseline to follow‐up, and a sample size calculation for a paired samples *t*‐test with an estimated effect size of 0.6, alpha 0.05, and power of 80% yielded an estimated 24 healthy subjects needed. A total of 30 subjects were enrolled to account for dropouts, non‐responders to DOMS, or lack of compliance with the TSD protocol.

All data are presented as means (± standard deviation [SD]) unless otherwise stated. Paired samples *t*‐tests were used to investigate any potential changes in QST measure and questionnaire scores before and after the intervention. Linear regression with backward selection was used to determine whether baseline QST and questionnaires could predict DOMS pain intensity after 24 and 48 h, respectively. Backward selection was used to remove the least contributing independent variables in each iteration and identify the best possible model. Assumptions were checked using appropriate statistical and visual methods. Statistical analysis was performed in the Statistical Package for the Social Sciences (SPSS), version 29.(IBM SPSS Statistics for Windows) and significance was accepted at *p* < 0.05.

## 3 RESULTS

### 3.1 Participant characteristics

The 30 participants (17 female) had a mean (SD) age of 23.1 (2.3) years and a mean (SD) body mass index (BMI) of 24.1 (5.7) kg/m^2^. All participants were compliant with the TSD protocol, which was confirmed by email, wrist actigraphy, or both. There was no missing data. See Table 1 for an overview of baseline factors.

**TABLE 1:** The 30 participants’ characteristics.

Baseline factors	Mean (±SD)
Demographics	
Age (years)	23.1 (±2.3)
BMI (kg/m^2^)	24.1 (±5.7)
Gender (female/male/other)	17/13/0
Inflammatory markers	
IL‐6 (pg/mL)	0.016 (±0.1)
Psychological and cognitive factors	
PCS	13.0 (±7.4)
PANAS negative	17.8 (±4.2)
PANAS positive	33.4 (±5.5)
PSQI	6.2 (±2.1)
4DSQ distress	6.9 (±5.1)
4DSQ depression	0.3 (±1.3)
4DSQ anxiety	1.6 (±2.3)
4DSQ somatization	6.6 (±5.1)
Quantitative sensory testing	
cPPT (kPa)	31.0 (±11.4)
cPTT (kPa)	87.0 (±13.8)
TSP (ΔkPa)	1.5 (±1.8)
CPM (ΔkPa)	12.1 (±17.5)

Abbreviations: BMI, body mass index; CPM, conditioned pain modulation; cPPT, cuff pressure pain detection threshold; cPTT, cuff pressure tolerance threshold; 4DSQ, Four‐Dimensional Symptom Questionnaire; IL‐6, interleukin 6; PANAS, Positive and Negative Affective Schedule; PSQI, Pittsburgh Sleep Quality Index; TSP, temporal summation of pain.

[Correction added on 20 February 2025, after first online publication: Values in Table 1 have been corrected.]

### 3.2 Intervention characteristics

The sleep quality (VAS 0–100) was significantly different between baseline, follow‐up, and the home rating (*F*[1.6, 46.0] = 230.3, *p* < 0.001). The participants all reported a mean (SD) sleep quality (VAS 0–100) of 73.2 (3.0) at baseline, which was then significantly lowered to 1.2 (0.7) following the TSD (*p* < 0.001), and then significantly increased to 73.5 (3.8) after the habitual sleep (*p* < 0.001). Furthermore, the level of rest was significantly different between baseline, follow‐up, and the home rating (*F*[2, 58] = 91.8, *p* < 0.001). The mean (SD) reported level of rest was 73.0 (2.9) at baseline, which was then significantly lowered to 17.5 (3.4) following the TSD (*p* < 0.001), and significantly increased to 66.9 (3.7) after the habitual sleep (*p* < 0.001).

The pain reported during flexing of the DOMS‐induced muscle significantly increased over the 48 h (*F*[2, 58] = 73.6, *p* < 0.001). The mean (SD) pain score was 5.8 (1.7) immediately at baseline (0 h), and then significantly increased at follow‐up (24 h) to 39.5 (4.3) (*p* < 0.001), and further increased at the home rating (48 h) to 55.9 (4.7) (*p* < 0.001).

### 3.3 Effect on psychological factors

The mean (SD) pain catastrophising score was 13.0 (7.4) at baseline (0 h) and significantly lowered to 10.1 (6.7) at follow‐up (24 h) (t_29_ = 2.3, *p* = 0.027). The mean (SD) PANAS positive affective scores were 33.4 (5.5) at baseline (0 h) and lowered to 29.1 (6.3) at follow‐up (24 h) (t_29_ = 6.5, *p* < 0.001). Furthermore, the mean (SD) PANAS negative affective scores were 17.8 (4.2) at baseline (0 h) and lowered to 15.9 (4.3) at follow‐up (24 h) (t_29_ = 3.2, *p* = 0.003). Finally, the mean (SD) 4DSQ anxiety domain score was 1.6 (2.3) at baseline (0 h) and 1.0 (1.7) at follow‐up (24 h) (t_29_ = 2.7, *p* = 0.012). There were no significant changes in any other measured psychological factors.

### 3.4 Effect on inflammatory marker

Plasma concentration of IL‐6 did not show significant differences when comparing its levels between baseline and follow‐up.

### 3.5 Effect on measures of pain sensitisation

The participants’ mean (SD) cPTT was 87.0 (13.8) kPa at baseline (0 h) and significantly lowered to 80.3 (17.4) kPa at follow‐up (24 h) (t_29_ = 2.7, *p* = 0.012). Furthermore, the mean (SD) CPM effect was 12.1 (17.5) at baseline (0 h) and significantly lowered to 5.3 (11.1) at follow‐up (24 h) (t_29_ = 2.2, *p* = 0.036). There were no changes in any other pain sensitivity measures.

### 3.6 Prediction of DOMS pain after TSD (24 h)

The initial model included demographics and baseline (0 h) measurements of inflammation, psychological factors, and pain sensitivity (Table 2) and explained 66% (*F*[6, 13] = 1.6, *p* > 0.05; Table 2) of the variability in DOMS pain after TSD (follow‐up, 24 h) with an adjusted *R* ^2^ of 25%. After nine iterations the final model included sex, BMI, IL‐6, PCS, PANAS positive and negative, DSQ somatisation, and cPPT and explained 58% (*F*[8, 21] = 3.6, *p* = 0.008; Table 2) of the variability in DOMS pain after TSD (24 h) with an adjusted *R* ^2^ of 42%.

**TABLE 2:** Initial and final linear regression model statistics for prediction of delayed onset muscle soreness pain after 24 h.

Iteration	Independent variable	β	*p*	*R* ^2^	Adjusted *R* ^2^
**1**				**0.66**	**0.25**
	Constant		<0.001		
	Age	−0.177	<0.001		
	Sex	−0.376	<0.001		
	BMI	−0.350	0.609		
	IL‐6	0.609	0.403		
	PCS	0.403	0.512		
	PANAS positive	0.512	<0.001		
	PANAS negative	−0.244	<0.001		
	PSQI	−0.091	<0.001		
	4DSQ distress	−0.169	<0.001		
	4DSQ depression	−0.043	0.027		
	4DSQ anxiety	0.027	<0.001		
	4DSQ somatisation	−0.341	<0.001		
	cPPT	−0.523	0.186		
	cPTT	0.186	0.105		
	TSP	0.105	<0.001		
	CPM	−0.053	0.821		
**9**				**0.58**	**0.42**
	Constant		0.004		
	Sex	−0.416	0.010		
	BMI	−0.393	0.023		
	IL‐6	0.513	0.003		
	PCS	0.445	0.023		
	PANAS positive	0.462	0.019		
	PANAS negative	−0.369	0.050		
	4DSQ somatisation	−0.309	0.071		
	cPPT	−0.464	0.017		

Abbreviations: BMI, body mass index; CPM, conditioned pain modulation; cPPT, cuff pressure pain detection threshold; cPTT, Cuff pressure tolerance threshold; 4DSQ, Four‐Dimensional Symptom Questionnaire; IL‐6, interleukin‐6; PANAS, Positive and Negative Affective Schedule; PCS, Pain Catastrophizing Scale; PSQI, Pittsburgh Sleep Quality Index; TSP, temporal summation of iain.

### 3.7 Prediction of DOMS after habitual sleep (48 h)

The initial model included demographics and baseline (0 h) measurements of inflammation, psychological factors, and pain sensitivity (Table 3) and explained 78.6% (*F*[16, 13] = 3.0, *p* = 0.026; Table 3) of the variability in DOMS pain at the home rating (48 h). After nine iterations the final model included BMI, IL‐6, PCS, PANAS positive, DSQ distress and depression, cPPT, and TSP and explained 68% (*F*[8, 21] = 5.7, *p* < 0.001; Table 3) of the variability in DOMS pain after habitual sleep with and adjusted *R* ^2^ of 56%.

**TABLE 3:** Initial and final linear regression model statistics for prediction of delayed onset muscle soreness pain after 48 h.

Iteration	Independent variable	β	*p*	*R* ^2^	Adjusted *R* ^2^
**1**				**0.79**	**0.52**
	Constant		0.012		
	Age	−0.092	0.634		
	Sex	0.050	0.780		
	BMI	−0.838	<0.001		
	IL‐6	0.340	0.070		
	PCS	0.501	0.027		
	PANAS POSITIVE	0.495	0.014		
	PANAS NEGATIVE	−0.164	0.424		
	PSQI	−0.265	0.198		
	DSQ distress	−0.427	0.172		
	DSQ depression	0.303	0.218		
	DSQ anxiety	0.108	0.608		
	DSQ somatisation	0.049	0.757		
	cPPT	−0.506	0.032		
	cPTT	−0.339	0.136		
	TSP	0.212	0.161		
	CPM	−0.191	0.315		
**9**				**0.68**	**0.56**
	Constant		0.003		
	BMI	−0.651	<0.001		
	IL‐6	0.462	0.002		
	PCS	0.299	0.044		
	PANAS POSITIVE	0.561	0.001		
	4DSQ distress	−0.442	0.026		
	4DSQ depression	0.316	0.097		
	cPPT	−0.703	<0.001		
	TSP	0.223	0.113		

Abbreviations: BMI, body mass index; CPM, conditioned pain modulation; cPPT, cuff pressure pain detection threshold; cPTT, cuff pressure tolerance threshold;4DSQ, Four‐Dimensional Symptom Questionnaire; IL‐6, interleukin‐6; PANAS, Positive and Negative Affective Schedule; PCS, Pain Catastrophizing Scale; PSQI, Pittsburgh Sleep Quality Index; TSP, temporal summation of pain.

## 4 DISCUSSION

This study demonstrated that the combination of 24‐h TSD and DOMS induced hypersensitivity to pain, impaired CPM, lowered positive and negative affective scores, lowered pain catastrophising scores, and reduced anxiety. Furthermore, sex, BMI baseline IL‐6, PCS, positive PANAS, and cPPT were independent predictors explaining 58% of the variance of DOMS pain intensity 24 h after initiation. Similarly, BMI and baseline IL‐6, PCS, positive PANAS, distress, and cPPT were independent predictors of 68% of DOMS pain 48 h after initiation.

### 4.1 Developing a new experimental pain model

The participants in the present study had a lowered quality of sleep, lowered level of rest, and mild‐to‐moderate pain following the TSD and DOMS intervention. TSD (Staffe et al., 2019) and sleep fragmentation (Simpson et al., 2018; Smith et al., 2007) have been demonstrated to increase sensitivity to various noxious stimulation modalities in healthy subjects, with impacts on measures of the peripheral and central pain mechanisms (Hertel et al., 2023; Smith et al., 2007; Staffe et al., 2019). DOMS evoked by standardised protocols induce movement‐evoked pain (McPhee & Graven‐Nielsen, 2018) varying from slight stiffness to severe and restrictive pain (Cheung et al., 2003) with localised pressure hyperalgesia (Kristensen et al., 2021), similar to the present study.

Previously, the effect of DOMS on TSP has been debated with reports of both increased facilitation (Nie et al., 2006; Torisu et al., 2010) and no changes (Kristensen et al., 2021) in line with the present study. Similarly, discrepancies exist regarding changes in TSP following experimental sleep provocations ranging from facilitation (Hertel et al., 2023; Staffe et al., 2019) to no changes (Schuh‐Hofer et al., 2013). CPM was impaired following the intervention in the present study but has previously been demonstrated to be stable during DOMS (Kristensen et al., 2021), emphasising the lack of central changes associated with DOMS models. Thus, the present work demonstrates that DOMS combined with TSD increases the comparability of the model to chronic pain conditions by mimicking this central change observed in subgroups of patients with severe chronic pain characterised by increased TSP and impaired CPM (Arendt‐Nielsen et al., 2015). Supporting this, a recent study investigating a DOMS and TSD combination found indications of changes in the central pain mechanisms based on decreased distal PPTs and enlarged referred pain areas (Palsson et al., 2023).

Increases in systemic inflammatory markers can be a consequence of both experimental sleep provocations (Irwin et al., 2016) and acute muscle injuries such as DOMS (MacIntyre et al., 2001). Prostaglandins and IL‐6 are known sensitisers of the peripheral nociceptors and mediate pain processing at the spinal level (Kawasaki et al., 2008), which can increase pain sensitivity. The present model was unable to demonstrate significant differences in IL‐6, which could be explained by the choice of sleep deprivation protocol, as a systematic review and meta‐analysis suggest that several days with sleep fragmentation provokes increased levels of IL‐6 when compared to a single night of TSD (Irwin et al., 2016). Further, IL‐6 has previously been demonstrated to increase within the first 6 h after DOMS induction and then decrease at the 24‐h mark (MacIntyre et al., 2001), being outside the blood test window for the present study.

Finally, the present study demonstrated changes in various psychological factors following the induction of DOMS and TSD. Poor quality of sleep has been suggested to exert pronociceptive effects through impairment of the emotional descending modulation of perceived pain (Huber et al., 2022), and recent animal studies have revealed that affective state transitions related to acute sleep loss might be mediated by dopamine release in the brain (Wu et al., 2024). Supporting this, experimental sleep provocations have previously been demonstrated to impact affective state, and positive affect has been found to be lowered after sleep fragmentation (Hertel et al., 2023), TSD (Stenson et al., 2021; Talbot et al., 2010) and partial sleep deprivation (Finan et al., 2017). This is similar to the present study, in which positive affect was lowered. Contrary to the present results, previous studies report either increases (Stenson et al., 2021) or no changes in negative affect (Finan et al., 2017; Hertel et al., 2023; Talbot et al., 2010). Similarly, both pain catastrophising and anxiety scores were lowered following the intervention contrary to expectations. Previous evidence suggests that poor quality of sleep in patients with chronic pain is associated with increased pain catastrophising (Boye Larsen et al., 2021). However, another recent study of healthy participants also reported puzzling directions of associations between pain catastrophising and sleep variables (Karmann et al., 2018). These conflicting findings indicate that future research is warranted to establish the direction of associations between sleep and psychological factors.

The rationale behind this study was to develop an experimental pain model that mimics chronic pain in terms of increased inflammation, increased pain sensitivity, and worsened psychological factors. The present study did find increased pain sensitivity but was unable to increase inflammation and psychological factors. However, some of the psychological changes observed were contradictory to current knowledge, and this needs to be further studied in larger and more diverse samples to establish whether this is a true effect or if subgroups might exist. The present model was unable to demonstrate changes in IL‐6, and future studies could attempt to explore if sleep fragmentation is better than a TSD model, as previous literature might support this (Irwin et al., 2016).

### 4.2 Prediction of pain following the new experimental pain model

A review has established that women report higher clinical pain intensities compared to men (LeResche, 2011), while sex differences in experimental pain are more unclear and modality specific (Racine et al., 2012). The present results indicated that female sex was predictive of increased DOMS pain at the 24‐h mark. Sex effects on DOMS pain have previously been disputed with reports of no differences (Lemos et al., 2023) and females reporting less pain (Dannecker et al., 2012). The conflicting results of the present study might be due to the effect of sleep deprivation on pain being moderated by sex, with females being more impacted (Racine et al., 2012). Higher BMI has previously been correlated with loss of strength and soreness (Kim & So, 2018) after exercise and has been suggested as a potential mediator of muscle damage after exercise. However, in the present study higher BMI was predictive of less DOMS pain after both 24 and 48 h. This might be because BMI does not take body fat mass and muscle into consideration and body composition has been suggested to be crucial for the extent of exercise‐induced muscle damage (Hickner et al., 2001). Future studies should aim to include more nuanced measures of body composition.

Several markers of psychological state have been identified as contributors to the pain experience and as potential mediators of the relationship between sleep and pain including mood, depression, and anxiety (Hertel et al., 2024; Kristensen et al., 2021). The present study identified higher baseline positive PANAS as an independent predictor of increased DOMS pain at both the 24‐ and 48‐h marks in contrast to a previous DOMS study that found the opposite tendency (Kristensen et al., 2021). Patients with chronic pain and poor quality of sleep report higher levels of pain catastrophising, anxiety, and depression compared to those with good sleep (Boye Larsen et al., 2021). Consistent with previous research suggesting pain catastrophising to be one of the most consistent predictors of adverse pain experiences explaining both variance in current pain and pain after treatment (Hertel et al., 2024), a higher PCS score was identified as an independent predictor of increased DOMS pain at both the 24‐ and 48‐h marks.

Finally, higher baseline IL‐6 emerged as a significant predictor of increased DOMS pain, which could indicate that healthy individuals with a more pronounced baseline inflammatory drive might be more susceptible to muscle pain and sleep loss. This might be attributed to a more sensitised pain system (Kawasaki et al., 2008), which would contribute to higher pain intensities. The present study found higher baseline cPPT to be predictive of less DOMS pain at both the 24‐ and 48‐h marks, indicating that less pain‐sensitive individuals had less intense pain. Previously, increased baseline pain sensitivity has been identified as a predictor of DOMS pain intensity (Kristensen et al., 2021).

### 4.3 Limitations and methodological considerations

This study was not designed to demonstrate separate effects of DOMS and TSD, preventing definitive conclusions on any potential additive effects, as was suggested in a recent study (Palsson et al., 2023). Although TSD was employed, previous research indicates that sleep continuity disturbances might have greater impacts on pain sensitivity (Finan et al., 2013) and inflammation (Irwin et al., 2016). Nevertheless, TSD provides a faster and more convenient method to impact the central pain mechanisms, which was deemed to outweigh possible smaller effects. The study recruited healthy young individuals who were predominantly university students, which might not reflect the entire population of healthy young adults as defined by in‐ and exclusion criteria. Future studies should strive to recruit a sample with more diverse socioeconomic backgrounds. Furthermore, the mean PSQI scores of the sample were above the defined cut‐off for significant sleep disturbance of >5, which might reduce the possible impact of the TSD. The model should be tested in a sample of healthy individuals with good baseline sleep to ensure a more homogenous group before the intervention. This study utilised linear regression to explore factors impacting pain development following TSD and DOMS. The initial model's extensive independent variables may result in overfitting and restrict generalisation due to a possibly inadequate sample size. Nevertheless, all collinearity levels were <0.1 and all variance inflation factor levels were <10. The present study could be methodologically limited, as it only assessed a single inflammatory marker (IL‐6). Additionally, a fixed battery of questionnaires was used. Future studies could examine if larger inflammatory panels and other psychological questionnaires, such as the Hospital Anxiety and Depression Scale or World Health Organisation‐Five Well‐Being Index questionnaire for psychological well‐being, could yield different results.

## 5 CONCLUSION

The present study successfully implemented a combined experimental pain model with DOMS and TSD with changes to peripheral and central pain mechanisms and psychological implications, resembling clinical musculoskeletal pain more closely. The model was unable to demonstrate changes in IL‐6. Further research is needed to investigate the impacts on psychological factors and determine whether the model could be a useful translational model of chronic pain. Baseline parameters explained 58% of DOMS pain after 24 h and 68% of DOMS pain after 48 h. However, the regression models were characterised by increased TSP and impaired CPM explorative and should be interpreted with care.

## AUTHOR CONTRIBUTIONS

**Emma Hertel:** Conceptualization; methodology; formal analysis; writing – review and editing; writing – original draft; supervision; data curation; validation; visualization. **Elaxmi Sathiyalingam:** Writing – review and editing; investigation; data curation. **Linea Pilgaard:** Writing – review and editing; investigation; data curation. **Simone Juline Brommann:** Data curation; writing – review and editing; investigation. **Rocco Giordano:** Conceptualization; data curation; writing – review and editing; formal analysis; supervision; investigation; methodology; validation. **Kristian Kjær‐Staal Petersen:** Conceptualization; methodology; formal analysis; supervision; funding acquisition; project administration; writing – original draft; writing – review and editing; validation; resources.

## CONFLICT OF INTEREST STATEMENT

All authors declare that they have no conflicts of interest.

## Data Availability

The data that support the findings of this study are available from the corresponding author upon reasonable request.
